# Serum lipidome associates with neuroimaging features in patients with traumatic brain injury

**DOI:** 10.1016/j.isci.2024.110654

**Published:** 2024-08-03

**Authors:** Ilias Thomas, Virginia F.J. Newcombe, Alex M. Dickens, Sophie Richter, Jussi P. Posti, Andrew I.R. Maas, Olli Tenovuo, Tuulia Hyötyläinen, András Büki, David K. Menon, Matej Orešič, CENTER-TBI MR subgroup Participants and Investigators

**Affiliations:** 1 School of Medical Sciences, Faculty of Medicine and Health, Örebro University, Örebro, Sweden; 2 School of Information and Engineering, Dalarna University, 79131 Falun, Sweden; 3 Division of Anaesthesia, Department of Medicine, University of Cambridge, Cambridge, UK; 4 Turku Bioscience Centre, University of Turku and Åbo Akademi University, Turku, Finland; 5 Department of Chemistry, University of Turku, Turku, Finland; 6 Neurocenter, Department of Neurosurgery and Turku Brain Injury Center, Turku University Hospital and University of Turku, Turku, Finland; 7 Department of Neurosurgery, Antwerp University Hospital and University of Antwerp, Edegem, Belgium; 8 Neurocenter, Department of Neurology and Turku Brain Injury Center, Turku University Hospital and University of Turku, Turku, Finland; 9 School of Science and Technology, Örebro University, Örebro, Sweden

**Keywords:** Neuroscience, Systems biology, Lipidomics

## Abstract

Acute traumatic brain injury (TBI) is associated with substantial abnormalities in lipid biology, including changes in the structural lipids that are present in the myelin in the brain. We investigated the relationship between traumatic microstructural changes in white matter from magnetic resonance imaging (MRI) and quantitative lipidomic changes from blood serum. The study cohort included 103 patients from the Collaborative European NeuroTrauma Effectiveness Research in TBI (CENTER-TBI) study. Diffusion tensor fitting generated fractional anisotropy (FA) and mean diffusivity (MD) maps for the MRI scans while ultra-high-performance liquid chromatography quadrupole time-of-flight mass spectrometry was applied to analyze the lipidome. Increasing severity of TBI was associated with higher MD and lower FA values, which scaled with different lipidomic signatures. There appears to be consistent patterns of lipid changes associating with the specific microstructure changes in the CNS white matter, but also regional specificity, suggesting that blood-based lipidomics may provide an insight into the underlying pathophysiology of TBI.

## Highlights

• Significant associations observed between WM injury and circulating lipids post TBI
• Decreasing lipid concentrations are associated with higher MD and lower FA values
• Increasing severity of TBI is associated with higher MD and lower FA values
• Regional variations exist between DTI metrics and circulating lipid metabolites

## Graphical abstract

**Figure:**
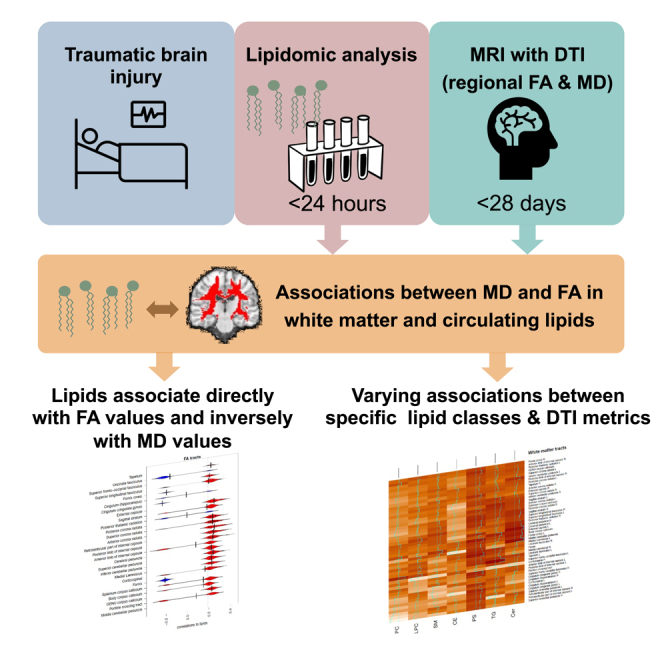

## Introduction

Traumatic brain injury (TBI) affects over 50 million people worldwide every year.^1^^,^^2^ Despite being so common, the dynamic pathophysiology and determinants of outcome trajectories remain poorly understood; TBI has been described as the most complex disease in the most complex organ.^3^ This is particularly true when attempting to understand the changes that may occur in circulating metabolites (including polar metabolites and lipids) after a TBI. Understanding these metabolic abnormalities is critical, since such knowledge might allow us to design and evaluate novel therapies. Indeed, we already know that TBI is associated with substantial metabolic abnormalities, which have been mainly demonstrated in humans using positron emission tomography^4^^,^^5^ and magnetic resonance spectroscopy.^6^^,^^7^ However, these techniques are expensive and logistically demanding.

Metabolomic and lipidomic analysis of blood provides one convenient approach to address this issue. We have previously reported extensive changes in the circulating metabolome and lipidome resulting from TBI, including changes proportional to disease severity and associated with patient outcomes.^8^^,^^9^^,^^10^ These metabolic changes provide evidence of the systemic impact of TBI and provide a means by which pathophysiological mechanisms in the brain may be explored by analysis of peripheral blood. One of the main metabolic changes we have observed following a TBI is changes in lipids,^10^ which are known to be present in cell membranes in neurons and glia and are a component of brain myelin. Myelin is rich in lipids which constitute approximately 80% of the dry weight of myelin, and this makes changes in lipid profile a prime target for characterizing damage to myelinated white matter. This is critical since white matter damage is an important driver of outcome in TBI.^11^

Magnetic resonance imaging (MRI) has the potential to characterize microstructural damage and improve our understanding of the pathophysiology underlying different lipidomic profiles after a TBI. In particular, advanced quantitative MRI, including diffusion tensor imaging (DTI), has been shown to be sensitive to injury after a TBI and has been associated with the severity of injury and outcome.^12^^,^^13^^,^^14^ DTI in particular is able to detect microstructural damage in the white matter, which may provide insights into the pathophysiology of injuries.^15^ Commonly used measures include fractional anisotropy (FA), a marker of white matter integrity, and mean diffusivity (MD), a measure of the magnitude of water diffusion, which reflects changes in cytotoxic and vasogenic edema acutely and neurodegeneration in the chronic phase. After TBI, changes in diffusivity have been found to be associated with longer-term functional outcome,^13^^,^^16^ and the trajectory of imaging changes detected are consistent with ongoing axonal degeneration.^15^^,^^17^ Blood levels of neurofilament light chain (NfL), a promising marker of this axonal degeneration, are able to predict the ongoing neurodegeneration detected on neuroimaging.^18^^,^^19^ This indicates that the structural changes seen on DTI and other advanced neuroimaging methods reflect pathophysiology that may be detectable using blood biomarkers.

Lipidomic profiling may provide more information about the molecular processes that underlie white matter integrity and, more generally, brain health. For example, circulating lipids can be utilized as a biomarker for microvascular brain disease.^20^ We have previously shown that the severity of TBI relates to the lipidomic signature in blood.^10^ In particular, choline phospholipids (lysophosphatidylcholines [LPCs], ether phosphatidylcholines [PCs], and sphingomyelins [SMs]) were inversely associated with TBI severity and were among the strongest predictors of patient outcomes.

Here we investigate the relationship between traumatic microstructural changes in the brain seen on MRI and quantitative lipidomic changes in the blood in a subset of patients recruited to the MRI sub-study of the Collaborative European NeuroTrauma Effectiveness Research in TBI (CENTER-TBI) study. Given that we have previously shown that the severity of TBI is associated with the lipidomic signature, here we hypothesized that the extent of injury detected using DTI would be associated with changes in lipid measures from the blood with regional specificity.

## Results

**Figure 1:**
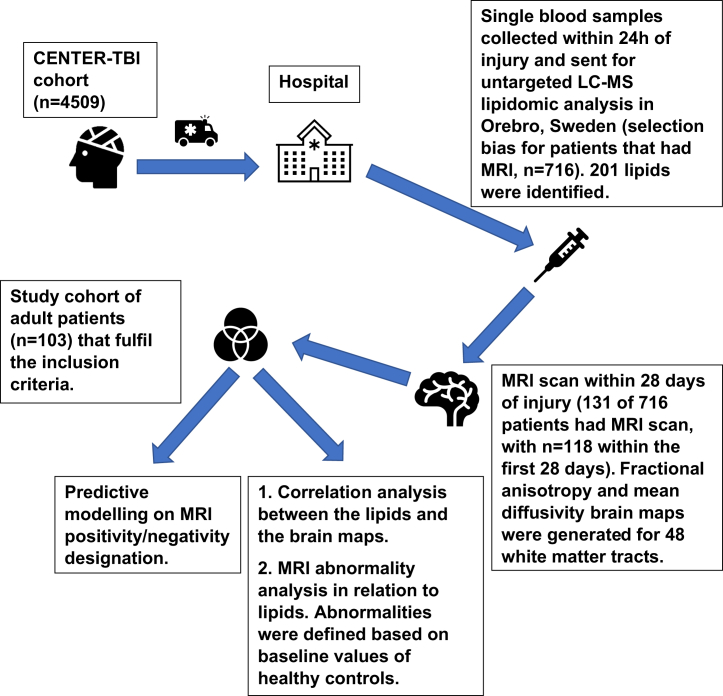
Flowchart of data acquisition and analysis The final analysis included 103 patients who fulfilled the inclusion criteria, and the classification models included 102. The 4,509 patients are the overall patient population of the CENTER-TBI study, and the 716 patients where the lipidomics analysis was performed is the same cohort as the main study population in Thomas et al.^10^ Not all patients from the CENTER-TBI study that had MRI scans are included in the 716 patients. In total, 131 of those 716 patients had MRI scans available. The inclusion criteria for the study were a clinical diagnosis of TBI, presentation to one of the 65 centers within 24 h of injury, MRI scanning done within 28 days from injury (118 of 131 patients), and being at least 18 years old (103 of 118 patients). Informed consent was obtained from all study participants or their legal representatives/next of kin, according to the local regulations of each center. The presence of severe, pre-existing neurological disorders was an exclusion criterion.

103 patients in CENTER-TBI had both an MRI scan within 28 days post-injury and blood samples available for lipidomic analysis within 24 h post-injury. The demographics of the study patient population and healthy controls in which MRI was performed can be seen in Table 1, and the study workflow is shown in Figure 1. In Table 1, we also provide details of a control group, which was the population used to harmonize the patient images across the sites where MRIs were taken. No lipidomic analysis was done in these control subjects. The majority of patients were male (TBI: 75%) with a median age of 43 (range 18–82) years. The median Glasgow Coma Score (GCS) score was 15 (interquartile range 11–15), and Glasgow Outcome Scale Extended (GOSE) score was 7 (interquartile range 6–8).

**Table 1:** Patient and control demographic characteristics

	Patients with TBI	Healthy controls
Number of participants	103	104
Age (years)^a^	43 (28.5–58)	39.5 (28.5–58)
Sex	77M/26F	61M/42F (1 missing)
Baseline GCS^a^	15 (11–15)	–
	68.9% mild TBI (GCS 13–15)	
	6.8% moderate TBI (GCS 9–12)	
	21.4% severe TBI (GCS ≤8)	
Stratum	33 admission (32 mild, 1 moderate)	
	28 ER (27 mild, 1 NA)	
	42 ICU (23 mild, 6 moderate, 22 severe, 2 NA)	
GOSE^a^	7 (6–8)	–
Time from injury to MRI scan (hours)^a^	54.9 (35.2–303.2)	–
MRI	62 positive/40 negative, 1 NA	–
CT	54 positive/45 negative, 4 NA	–

CT, computed tomography; ER, emergency room; GCS, Glasgow Coma Score; GOSE, Glasgow Outcome Scale Extended; ICU, intensive care unit; MRI, magnetic resonance imaging.

a Median (interquartile range).

For the baseline MRI findings, there were 102 patients in the dataset that had an MRI report of visible lesions available, 62 of which had a visible intracranial lesion (MRI positive). For the baseline CT scan, 45 had no intracranial lesion detectable on CT (CT negative). As it is known that CT-negative patients may still have long-term sequelae after TBI^21^ and CT may miss lesions visible on MRI^22^^,^^23^ (in this analysis 11 of 45 were MRI positive), a separate model was developed for this subgroup of patients (*n* = 45).

DTI images were used to define 48 regions of interest (ROIs) for the white matter tracts and extract FA and MD values (Table S1; Figure S1). In a separate analysis, segmentation of T1-weighted images was applied to calculate volumetric data for 51 ROIs (Table S2).

Following lipidomic analysis, 201 known lipids were quantified that belong to the major lipid functional groups including ceramides (Cer), LPCs, PCs, SMs), cholesterol esters (CEs), and triacylglycerols (TGs).

### Classification models can detect patients with brain abnormalities based on lipidomic profiles

Penalized logistic regression models confirmed the discriminatory ability of the lipids for MRI positivity/negativity discrimination for all patients, with area under the curve (AUC) of 0.83–0.85 (Table S3). Furthermore, for the subset of patients with negative CT, it was still possible to classify the patients with positive MRI, with lower AUC (0.70). All models had MRI positivity/negativity as dependent variable and the lipid concentrations as independent variables.

### Circulating lipidome associates with neuroimage findings and burden of injury

Overall, the results show that specific lipid classes are correlated with the findings of MRI scans. Patients showed reduced FA values and increased MD values when compared to controls. FA abnormalities in patients mostly correlated with PCs and LPCs, and MD mostly with SMs and PCs. The top 25 lipids for each set are shown in Table 2. In general, FA values have positive correlations with the lipids, while MD have negative (Tables S4 and S5). Positive correlation in this context means that the FA and lipid values move in the same direction, i.e., both are reduced. Negative correlation means that MD and lipid values move in opposite directions. For the volumetric dataset, the classes that were overwhelmingly represented in significant correlations were PC, LPC, and TG (Table 2), with changes in the volume of ROIs in patients with reference to control data showing variable relationships to lipid levels (Table S6).

**Table 2:** Lipids that have the largest relative frequencies (number of correlations divided by the number of ROIs) of significant correlation to the neuroimaging sets

Associations with FA		Associations with MD		Associations with volume	
lipid [identification level]	Freq	lipid [identification level]	Freq	lipid [identification level]	Freq
LPC(20:4) [1]	0.479	PC(O-36:4) [2]	0.354	PC(36:4) [2]	0.431
LPC(16:0) [1]	0.417	PC(O-38:4) [2]	0.333	TG(16:0/18:2/18:2) [2]	0.431
LPC(20:5) [2]	0.312	SM(d18:2/18:1) [2]	0.312	TG(54:6) [2]	0.373
LPC(16:0e) [2]	0.292	PC(O-22:1/20:4) [2]	0.271	CE fragment [3]	0.353
LPC(18:1) [1]	0.292	PC(34:3) [2]	0.250	PC(O-38:6) [2]	0.294
PC(34:3) [2]	0.292	PC(O-38:5) [2]	0.250	TG(18:2/22:5/16:0) [2]	0.216
PC(35:1) [2]	0.292	SM(d18:1/22:1) [2]	0.250	PC(O-40:6) [2]	0.196
PC(O-36:4) [2]	0.292	SM(d41:2) [2]	0.250	TG(54:6) [2]	0.196
PC(O-38:5) [2]	0.292	PC(O-40:6) [2]	0.229	PC(33:0) [2]	0.176
PC(O-40:6) [2]	0.292	TG (48:4) [2]	0.229	SM(d18:1/24:0) [2]	0.176
LPC(18:2) [1]	0.271	PC(P-18:0/18:1) [1]	0.208	LPC(16:0) [1]	0.157
PC(O-36:3) [2]	0.271	PC(O-40:5) [2]	0.208	LPC(16:0e) [2]	0.157
LPC(18:0) [1]	0.250	SM(d18:1/18:1) [1]	0.208	SM(d18:1/22:1) [2]	0.157
PC(36:4) [2]	0.250	PC(35:4) [2]	0.188	LPC(18:0) [1]	0.137
PC(40:8) [2]	0.250	SM(d16:1/23:0) [2]	0.188	LPC(20:5) [2]	0.137
PC(O-38:4) [2]	0.250	SM(d41:1) [2]	0.167	PC(38:1) [2]	0.137
SM(d36:2) [2]	0.250	TG(58:10) [2]	0.167	PC(O-40:5) [2]	0.137
TG (48:4) [2]	0.250	PC(36:4) [2]	0.146	PC(O-40:6) [2]	0.137
PC(35:4) [2]	0.208	SM(d40:1) [2]	0.146	PC(P-18:0/22:6) [1]	0.118
PC(37:4) [2]	0.208	PC(34:2) [2]	0.125	SM(d18:1/24:2) [2]	0.118
LPC(14:0) [2]	0.188	PC(O-36:3) [2]	0.125	LPC(22:6) [2]	0.098
LPC(22:6) [1]	0.188	SM(d18:1/24:0) [2]	0.125	TG (48:4) [2]	0.098
PC(O-34:2) [2]	0.188	SM(d38:2) [2]	0.125	LacCer(d18:1/16:0 [1])	0.078
PC(O-40:5) [2]	0.188	LPC(14:0) [2]	0.104	LPC(18:1) [1]	0.078
PC(P-18:0/18:2) [2]	0.188	PC(35:1) [2]	0.104	LPC(18:2) [1]	0.078

The reported lipids are from the same dataset as in previous study^10^ and were identified at the levels 1 or 2 (as stated in the table) according to the Metabolomics Standards Initiative (see STAR methods).

The ROIs where DTI metrics showed the most frequent significant correlations (frequency ≥10%) with lipids (201 total lipids) are shown in Tables S4–S6, as frequencies of significant correlations to the lipids. The ROIs with the highest frequency of significant correlations between FA and lipids were the corona radiata and the cerebellar peduncle. For MD data, the corticospinal tract and the superior longitudinal fasciculus are the areas with the highest frequencies of significant correlations with lipid metabolite levels (Table S4).

**Figure 2:**
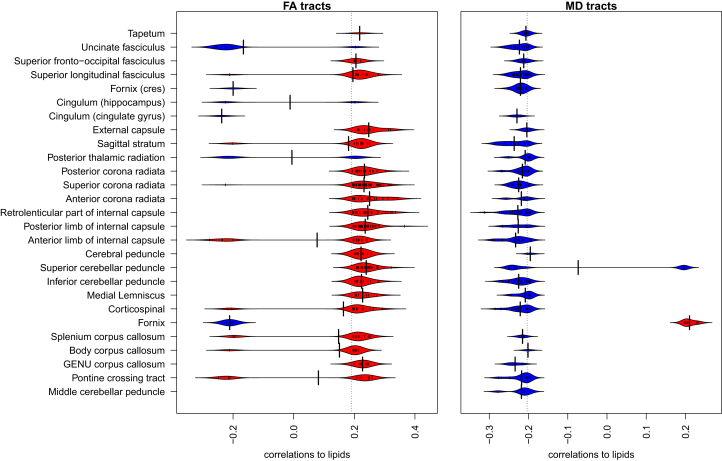
Correlations of the white matter tracts to individual lipid concentrations The beanpots of the correlation values between circulating lipid levels and DTI metrics show that fractional anisotropy (FA; left panel) shows mostly positive correlations with lipid levels (average R ∼0.2), while mean diffusivity (MD; right panel) shows mostly negative correlations with lipid levels (average R ∼0.2). Positive correlation in this context means that the FA and lipid values move in the same direction, i.e., both are reduced. Negative correlation means that MD and lipid values move in opposite directions. All lipids were included in the calculations, and the correlations were aggregated per tract.

Furthermore, for each ROI the values of the significant correlations were plotted as a beanplot. The correlations between lipid levels and FA and MD data can be seen in Figure 2. For FA, the correlations between ROIs and lipids are mostly positive, and for MD predominantly negative, similar to the volumetric data as seen in Figure S2 (also shown in Tables S4–S6).

**Figure 3:**
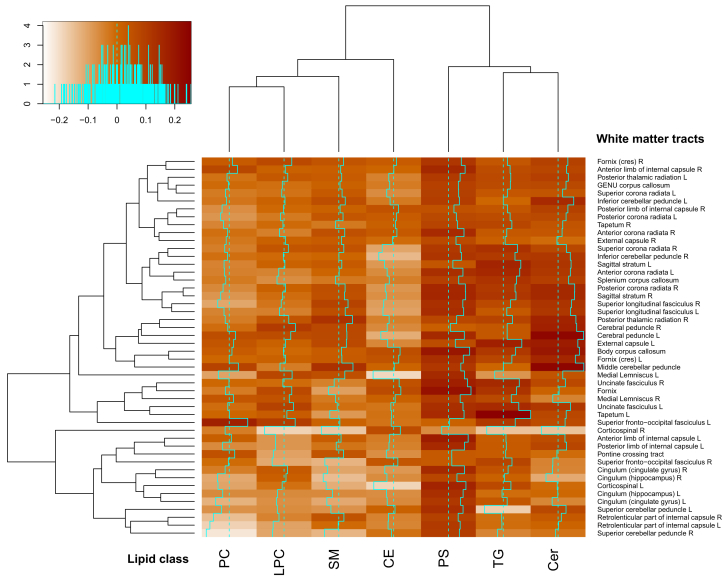
Relation of the main lipid groups to injury location The relationship between the lipidomic abnormalities and the frequency of injury in individual white matter tracts was examined in each tract across the study population. The top left panel shows the color key to the correlation values. The main matrix shows this analysis across all tracts and lipid classes. The color scale on the matrix shows correlations between individual white matter tracts (y axis) and lipid class (x axis). The dotted line shows a correlation of 0, and the solid line the correlation value for each lipid class in each region. The dendrograms on the X and Y axis show that the correlation patterns show clustering across the range of white matter tracts and lipid classes. (*The analysis was controlled for age and sex. A linear regression model for the controls was fitted for each ROI, and ROIs were defined as injured if both of the FA and MD values were outside the respective baseline range [one standard deviation] of healthy control values. The lipid group values are unadjusted summed concentrations of the lipids within each group. The lipid groups clusters were based on correlation analysis. Overall, PS, TG, and Cer have mostly positive correlations to the burden of injury, while CE, PC, SM, and LPC show mostly negative correlations. L, left; R, right*).

A heatmap that shows the lipid class values related to the burden of injury (defined as the percentages of ROIs that showed significant abnormalities in both FA and MD when compared to controls) in each of the 48 ROIs is shown in Figure 3. Overall, PS, TG, and Cer have mostly positive correlations to the burden of injury, while CE, PC, SM and LPC show mostly negative correlations meaning that lower concentration of SM, PC, and LPC is related to higher burden of injury. This is consistent with previous findings,^10^ where TBI severity was found to be associated with lower concentration of the same lipid groups.

### TBI location is not related to lipidomic patterns

**Figure 4:**
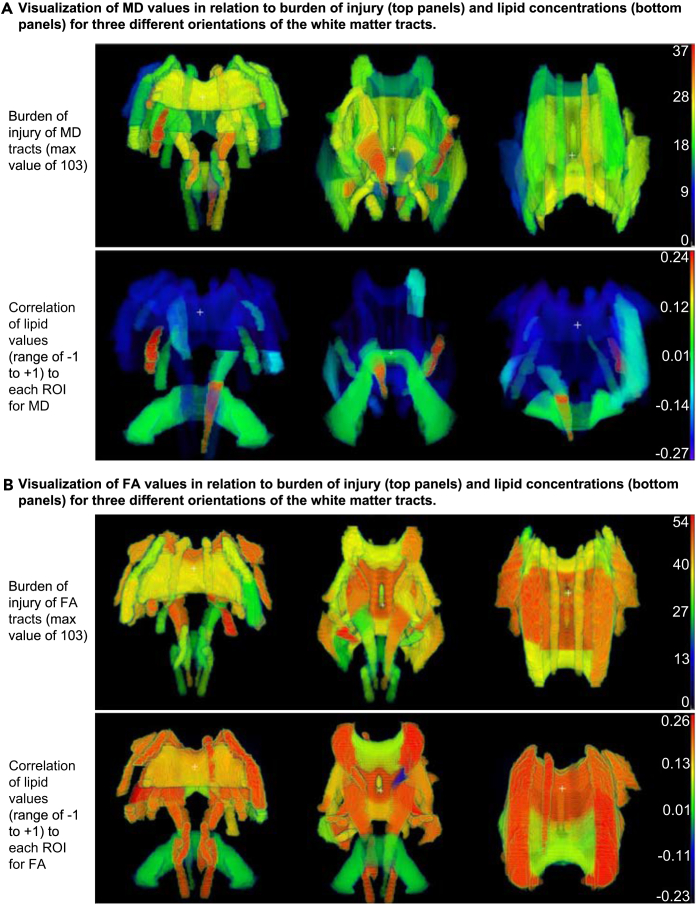
Brain maps of lipid correlations and burden of injury with three different brain orientations (three columns) The three orientations are the following (from left to right): anterior coronal, posterior coronal, and axial. (A) The upper panel shows the frequency of MD abnormalities in different white matter tracts across the 103 patients in the study. The cumulative frequencies depicted by color scale on the right have a maximum value of 103, the number of patients. A number of 37 would mean that 37 out of 103 patients showed abnormalities in the specific ROI. The lower panel visualizes the average value of significant lipid correlations with specific ROIs, between −1 and +1. All lipids that showed significant correlation with a specific ROI are included with their average correlation value shown. (B) Same analysis as in (A), but for FA data. Overall, no clear relationships were observed between regional lesion frequency and lipidomic abnormalities.

To evaluate if the high correlations of lipids with ROIs are related to the regional burden of injury, MD ROIs were mapped into the John Hopkins University (JHU) brain atlas,^24^ as shown in Figure 4A. The first row shows the frequency of MD abnormalities across the 103 patients in the study, while the second row visualizes the average value of significant lipid correlations with specific ROIs. Figure 4B shows the same analysis for FA data. Overall, no clear patterns were observed between regional lesion frequency and lipidomic abnormalities.

### Correlation networks show discrete interactions between lipid classes and white matter parcellation

Finally, the top 40 lipids that correlated the most with FA and the top 40 for the MD set were grouped into their respective lipid classes (extended from the top 25 shown in Table 2). For the FA and MD feature sets, the white matter tracts were parcellated into three classes: midline, left, and right; and correlation networks were explored between lipid sets and DTI metrics in these ROI classes. These correlations were controlled for the time elapsed between injury and blood sample draw, time between the injury and the MR scan, propofol administration, and age. Only significant partial correlations were included in the network, as defined by an alpha level of 0.05.

**Figure 5:**
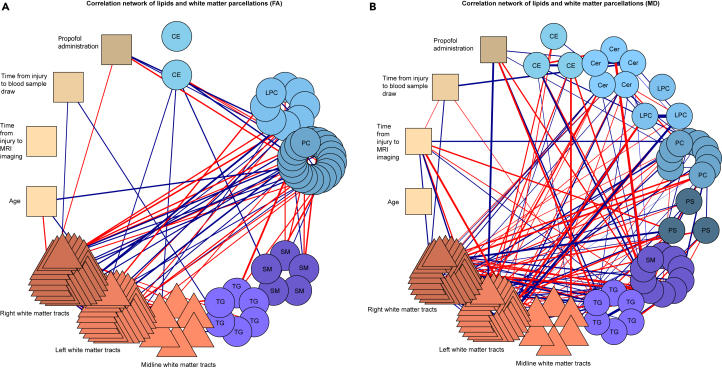
Partial correlation networks These networks display the individual lipid correlations to other lipids and the white matter tracts, controlled for time from injury to blood drawing, time from injury to MRI scan, age, and if propofol was administrated to the patients. Red edges indicate negative correlations and dark blue edges positive. The 40 lipids with the highest number of significant correlations are shown with all 48 white matter tracts. From top and clockwise the following groups are shown: CE, Cer, LPC, PC, PS, SM, and TG, followed by the white matter tracks split into three groups (midline, left, right) and individual variables of interest (age, time from injury to scan, time from injury to blood draw, and whether propofol was administered). For the lipids, each circle represents an individual lipid, colored by functional group, and for the white matter tracts each triangle represents one tract, colored by location. The four variables of interest are represented by squares. The functional groups of the lipids appear inside the circles (to the extent possible). The white matter tracts and the four control variables are denoted with text outside their respective shapes; the colored lines denote their relationships with brain regions and variables as described earlier. (A) Relationship with FA in white matter tracts. Overall, Cer and LPC have the highest correlation with FA in white matter tracts, and the timing of the samples, age, or propofol administration does not correlate with the lipids. (B) Relationship with MD in white matter tracts. More significant correlations can be seen here, compared to FA, and almost all groups have correlations with MD in white matter tracts. Abbreviations: CE, cholesterol ester; Cer, ceramide; LPC, lysophosphatidylcholine; PC, phosphatidylcholine; PS, phosphatidylserine; SM, sphingomyelin; TG, triacylglycerol; FA, fractional anisotropy measures; MD, mean diffusivity.

The results for FA are shown in Figure 5A. PC, LPC, SM, and TG classes all show strong correlations with the different white matter tracts. For the MD set, the most strongly related classes of lipids are PC and SM (Figure 5B). Lipid levels do not seem to be affected by the time elapsed between injury and blood sample collection nor the time of scans, when controlled for in the networks.

## Discussion

Following TBI, we show specific associations between FA and MD abnormalities on DTI imaging and the concentrations of circulating lipids. As expected, lower FA and higher MD values correlated with the severity of TBI and the magnitude of abnormality in lipids.^25^ We also observed that the associations between lipidomic abnormalities for FA and MD were different, suggesting different metabolic specificities for these two DTI metrics of white matter injury. LPCs associated mostly with changes in FA, while SMs associated with changes in MD. Only PCs showed strong associations with both DTI metrics and with volumetric data.

FA has been shown to depict changes in the microstructure of the brain.^26^ However, connecting FA changes to specific brain microstructure changes is challenging due to the biologically nonspecific nature of FA.^27^ The primary associations with FA in our data were found with LPCs. We found previously that decreased serum LPC concentrations associated with more severe disease and poorer outcomes in TBI.^10^ In animal models of TBI, LPC has been identified as one of the key lipid classes that increased in the CNS following the injury^28^ and correlated with the presence of MCP-1 in the hippocampus^29^—a key protein in attracting immune cells to the brain. However, none of these studies measured the LPCs concentrations in the blood. There are known transporters of LPCs in the CNS,^30^ and decreased levels of circulating LPC have been associated with poor outcomes in other CNS diseases.^31^ LPCs are also implicated in a wide range of inflammatory diseases.^32^ Given these previous findings, the positive associations of LPCs with FA as observed in our study may either reflect an increased inflammatory drive, or evidence of microstructural white matter disruption due to such inflammation (or other injury mechanisms).

SM is a key lipid class needed for the synthesis of myelin in the CNS.^33^ Decreased serum levels of SMs following a TBI and inverse associations with MD changes suggest that SMs may be recruited for myelin repair following the membrane damage in TBI. Similar inverse relationship between SMs levels and MD has been observed in Alzheimer’s disease.^34^ SMs are also converted to Cer in inflammatory conditions, which could be another non-exclusive cause for reduced levels of circulating SMs.

The lipids, which were consistently associated with both the diffusion imaging and the volumetric data, were PCs. PCs are a key component of cellular membranes, and therefore changes in brain microstructure will disrupt PC metabolism.^35^ PCs can also be broken down into free fatty acids, which can be utilized as an energy source in the CNS. Other acute brain injuries have been shown to increase activity of enzymes involved in fatty acid metabolism immediately following the injury.^36^ We have earlier shown that medium-chain fatty acids are associated with the severity of TBI.^9^^,^^10^ Furthermore, TBI causes an energy crisis within the CNS^37^ and therefore the increased utilization of PCs could be related to the severity of the energy crisis that is reflected in the poor imaging metrics.

The association with quantitative white matter imaging metrics and PCs was global as can be seen in Figure 3. This global distribution of association is seen to a lesser extent with the TGs. In contrast, the Cer associations seem to form two main clusters: one of more central fibers with the strongest associations and the other of the longer tracts with lower association. Cer and PCs associations were also stronger with these longer white tracts, while SMs were associated with more central tracts. The pathophysiology leading to these different predilections for different types and regions of white matter is likely to be complex. An increase in Cer in the plasma has been associated with several inflammatory and neurodegenerative diseases including multiple sclerosis^38^ and Alzheimer’s disease.^39^ The different roles Cer play in energy balance may influence different patterns seen.^40^

The observed lack of lipid associations with specific brain regions in the gray matter suggests that changes in the levels of these circulating lipids do not reflect the primary, non-contusion-related, cortical injury in TBI. In contrast, there were consistent associations with volumetric data from deeper white matter structures which may be driven by axonal damage. These findings are in line with our previous work, where we could see clear associations between polar metabolites and deeper brain volume changes.^41^ This suggests that the patterns of associations between the volumes and metabolites regardless of type are more clear in the deep white matter regions, rather than the cortical mantle. These regional differences in correlations with lipidomic signatures probably reflect changes in local cell types and are broadly in keeping with the heterogeneity in cellular lipid composition between dissociated cells from a single brain region (cerebellum).^42^

This is the largest study to date combining serum metabolomics and MRI imaging including the diffusion-weighted imaging. However, the sample size is still small and further validation studies are required to support our findings. The underlying reasons for the lipid changes observed are also difficult to pinpoint due to the difficulty in obtaining tissue or fluid samples from within the CNS, and therefore studies in suitable experimental models would be needed in order to understand the causes of the observed lipid changes.

In conclusion, we have identified groups of lipids which associate with specific MRI imaging metrics following TBI. There appears to be consistent patterns of lipid changes associating with the specific microstructure changes in the CNS white matter. There is also a pattern of lipids with regional specificity, suggesting that blood-based lipidomics may provide an insight into the underlying disease mechanisms in TBI.

### Limitations of the study

This was a multicenter study, and it was not possible to scan all patients within a specific time window after injury. Thus, the time difference between injury and imaging is considered a limitation of this study. Our time window for MRI ranged from one to 28 days post-injury. As the characteristics of traumatic intracranial findings evolve in this time frame, future studies should aim at determining optimal timing for MRI.^43^ This study did not investigate the association of lipidomic signature with TBI classification as it was investigated before.^10^ All data that were available were used for this study and may be underpowered, and as such it needs replicating with larger numbers.

## Consortia

### CENTER-TBI MR subgroup participants and investigators

Krisztina Amrein,^1^ Nada Andelic,^2^ Lasse Andreassen,^3^ Audny Anke,^4^ Philippe Azouvi,^5^ Bo-Michael Bellander,^6^ Habib Benali,^7^ Andras Buki,^8^ Alessio Caccioppola,^9^ Emiliana Calappi,^9^ Marco Carbonara,^9^ Giuseppe Citerio,^10, 11^ Hans Clusmann,^12^ Mark Coburn,^13^ Jonathan Coles,^14^ Marta Correia,^15^ Endre Czeiter,^8^ Véronique De Keyser,^16^ Vincent Degos,^7^ Bart Depreitere,^17^ Live Eikenes,^18^ Erzsébet Ezer,^19^ Kelly Foks,^20^ Shirin Frisvold,^21^ Damien Galanaud,^7^ Alexandre Ghuysen,^22^ Ben Glocker,^23^ Asta Haberg,^24, 25^ Iain Haitsma,^26^ Eirik Helseth,^27^ Peter J. Hutchinson,^28^ Evgenios Kornaropoulos,^29^ Noémi Kovács,^30^ Ana Kowark,^31^ Steven Laureys,^32^ Didier Ledoux,^32^ Hester Lingsma,^33^ Andrew I.R. Maas,^16^ Geoffrey Manley,^34^ David K. Menon,^29^ Tomas Menovsky,^16^ Benoit Misset,^32^ Visakh Muraleedharan,^35^ Ingeborg Nakken,^36^ Virginia Newcombe,^29^ Wibeke Nordhøy,^37^ József Nyirádi,^1^ Fabrizio Ortolano,^9^ Paul M. Parizel,^38^ Vincent Perlbarg,^7^ Paolo Persona,^39^ Wilco Peul,^40^ Jussi P. Posti,^41^ Louis Puybasset,^42^ Sophie Richter,^29^ Cecilie Roe,^43^ Olav Roise,^44,45^ Rolf Rossaint,^31^ Sandra Rossi,^39^ Daniel Rueckert,^23^ Ranjit D. Singh,^40^ Toril Skandsen,^24, 25^ Abayomi Sorinola,^46^ Emmanuel Stamatakis,^29^ Ewout W. Steyerberg,^33, 47^ Nino Stocchetti,^48^ Riikka Takala,^49^ Viktória Tamás,^46^ Olli Tenovuo,^41^ Zoltán Vámos,^19^ Gregory Van der Steen,^16^ Inge A. van Erp,^40^ Wim Van Hecke,^50^ Thijs Vande Vyvere,^50^ Jan Verheyden,^50^ Anne Vik,^24, 51^ Victor Volovici,^26^ Lars T. Westlye,^52^, Daniel Whitehouse,^29^ Guy Williams,^29^ Stefan Winzeck,^29^ Peter Ylén,^53^ Tommaso Zoerle^9^

^1^János Szentágothai Research Centre, University of Pécs, Pécs, Hungary.

^2^Division of Clinical Neuroscience, Department of Physical Medicine and Rehabilitation, Oslo University Hospital and University of Oslo, Oslo, Norway.

^3^Department of Neurosurgery, University Hospital Northern Norway, Tromso, Norway.

^4^Department of Physical Medicine and Rehabilitation, University Hospital Northern Norway, Tromso, Norway.

^5^Raymond Poincare hospital, Assistance Publique – Hopitaux de Paris, Paris, France.

^6^Department of Neurosurgery & Anesthesia & intensive care medicine, Karolinska University Hospital, Stockholm, Sweden.

^7^Anesthesie-Réanimation, Assistance Publique – Hopitaux de Paris, Paris, France.

^8^Department of Neurosurgery, Medical School, University of Pécs, Hungary and Neurotrauma Research Group, János Szentágothai Research Centre, University of Pécs, Hungary.

^9^Neuro ICU, Fondazione IRCCS Cà Granda Ospedale Maggiore Policlinico, Milan, Italy.

^10^School of Medicine and Surgery, Università Milano Bicocca, Milano, Italy.

^11^NeuroIntensive Care Unit, Department Neuroscience, IRCCS Fondazione San Gerardo dei Tintori, Monza, Italy.

^12^Department of Neurosurgery, Medical Faculty RWTH Aachen University, Aachen, Germany.

^13^Department of Anesthesiology and Intensive Care Medicine, University Hospital Bonn, Bonn, Germany.

^14^Department of Anesthesia & Neurointensive Care, Cambridge University Hospital NHS Foundation Trust, Cambridge, UK.

^15^Radiology/MRI department, MRC Cognition and Brain Sciences Unit, Cambridge, UK.

^16^Department of Neurosurgery, Antwerp University Hospital and University of Antwerp, Edegem, Belgium.

^17^Department of Neurosurgery, University Hospitals Leuven, Leuven, Belgium.

^18^Department of Circulation and Medical Imaging, Norwegian University of Science and Technology, NTNU, Trondheim, Norway.

^19^Department of Anaesthesiology and Intensive Therapy, University of Pécs, Pécs, Hungary.

^20^Department of Neurology, Erasmus MC, Rotterdam, the Netherlands

^21^Department of Anesthesiology and Intensive care, University Hospital Northern Norway, Tromso, Norway.

^22^Emergency Department, CHU, Liège, Belgium.

^23^Department of Computing, Imperial College London, London, UK.

^24^Department of Neuromedicine and Movement Science, Norwegian University of Science and Technology, NTNU, Trondheim, Norway.

^25^Department of Physical Medicine and Rehabilitation, St.Olavs Hospital, Trondheim University Hospital, Trondheim, Norway.

^26^Department of Neurosurgery, Erasmus MC, Rotterdam, the Netherlands

^27^Department of Neurosurgery, Oslo University Hospital, Oslo, Norway.

^28^Division of Neurosurgery, Department of Clinical Neurosciences, Addenbrooke’s Hospital & University of Cambridge, Cambridge, UK.

^29^Division of Anesthesia, University of Cambridge, Addenbrooke’s Hospital, Cambridge, UK.

^30^Hungarian Brain Research Program - Grant No. KTIA_13_NAP-A-II/8, University of Pécs, Pécs, Hungary.

^31^Department of Anaesthesiology, University Hospital of Aachen, Aachen, Germany.

^32^Cyclotron Research Center, University of Liège, Liège, Belgium.

^33^Department of Public Health, Erasmus Medical Center-University Medical Center, Rotterdam, The Netherlands

^34^Department of Neurological Surgery, University of California, San Francisco, California, USA

^35^Karolinska Institutet, INCF International Neuroinformatics Coordinating Facility, Stockholm, Sweden.

^36^Department of Radiology and Nuclear Medicine, St.Olavs Hospital, Trondheim University Hospital, Trondheim, Norway.

^37^Department of Diagnostic Physics, Clinic of Radiology and Nuclear Medicine, Oslo University Hospital, Oslo, Norway.

^38^Department of Radiology, University of Antwerp, Edegem, Belgium.

^39^Department of Anesthesia & Intensive Care, Azienda Ospedaliera Università di Padova, Padova, Italy.

^40^Dept. of Neurosurgery, Leiden University Medical Center, Leiden, The Netherlands and Dept. of Neurosurgery, Medical Center Haaglanden, The Hague, The Netherlands

^41^Division of Clinical Neurosciences, Department of Neurosurgery and Turku Brain Injury Centre, Turku University Hospital and University of Turku, Turku, Finland.

^42^Department of Anesthesiology and Critical Care, Pitié -Salpêtrière Teaching Hospital, Assistance Publique, Hôpitaux de Paris and University Pierre et Marie Curie, Paris, France.

^43^Department of Physical Medicine and Rehabilitation, Oslo University Hospital/University of Oslo, Oslo, Norway.

^44^Division of Orthopedics, Oslo University Hospital, Oslo, Norway.

^45^Institute of Clinical Medicine, Faculty of medicine, University of Oslo, Oslo, Norway.

^46^Department of Neurosurgery, University of Pécs, Pécs, Hungary.

^47^Dept. of Department of Biomedical Data Sciences, Leiden University Medical Center, Leiden, The Netherlands

^48^Department of Pathophysiology and Transplantation, Milan University, and Neuroscience ICU, Fondazione IRCCS Cà Granda Ospedale Maggiore Policlinico, Milano, Italy.

^49^Perioperative Services, Intensive Care Medicine and Pain Management, Turku University Hospital and University of Turku, Turku, Finland.

^50^icoMetrix NV, Leuven, Belgium.

^51^Department of Neurosurgery, St.Olavs Hospital, Trondheim University Hospital, Trondheim, Norway.

^52^Norwegian Centre for Mental Disorders Research (NORMENT), Division of Mental Health and Addiction, Oslo University Hospital and Institute of Clinical Medicine, University of Oslo and Department of Psychology, University of Oslo, Oslo, Norway.

^53^VTT Technical Research Centre, Tampere, Finland.

## STAR★Methods

### Key resources table

**Table:** 

REAGENT or RESOURCE	SOURCE	IDENTIFIER
**Biological samples**		

Serum samples	Thomas et al.^10^	N/A

**Chemicals, peptides, and recombinant proteins**		

LC-MS lipidomics protocol	Thomas et al.^10^	N/A
MRI protocol	https://www.center-tbi.eu/project/mri-study-protocols	N/A

**Deposited data**		

Raw and analyzed data	This manuscript	Data deposited in CENTER-TBI repository, access as described in ‘data and code availability’. Custom code deposited in Mendeley data: https://doi.org/10.17632/2gbs56tzpc.1

**Software and algorithms**		

R (v4.1.2)	https://www.r-project.org/	N/A
fsl	https://fsl.fmrib.ox.ac.uk	N/A
ANTS	http://stnava.github.io/ANTs/	N/A

### Resource availability

#### Lead contact

Further information and requests for resources and reagents should be directed to and will be fulfilled by the Lead Contact, Matej Orešič (matej.oresic@oru.se).

#### Materials availability

This study did not generate new unique reagents.

#### Data and code availability

• Data are accessible based on submission of a data access request through the CENTER-TBI website: https://www.center-tbi.eu/data. CENTER-TBI is committed to data sharing and to responsible further use of the data. Hereto, we have a data sharing statement in place: https://www.center-tbi.eu/data/sharing. The CENTER-TBI Management Committee, in collaboration with the General Assembly, established the Data Sharing policy and Publication and Authorship Guidelines to assure correct and appropriate use of the data as the dataset is hugely complex and requires help of experts from the Data Curation Team or Bio-Statistical Team for correct use. This means that we encourage researchers to contact the CENTER-TBI team for any research plans and the Data Curation Team for any help in appropriate use of the data, including sharing of scripts. The complete Manual for data access is also available online: https://www.center-tbi.eu/files/SOP-Manual-DAPR-20181101.pdf.
• This paper does not report original code, but custom code was developed. All analysis used libraries available in R. Codes are available on Mandeley data (Thomas, Ilias (2024), “TBI MRI-Lipidomics”, Mendeley Data, V1, https://doi.org/10.17632/2gbs56tzpc.1)
• Any additional information required to reanalyze the data reported in this paper is available from the lead contact upon request.

### Experimental model and study participant details

The CENTER-TBI study recruited 4509 patients from 18 European countries and Israel (https://www.center-tbi.eu/, registered at clinicaltrials.gov NCT02210221).^22^ The CENTER-TBI database contains data from 65 centers whose data were collected between December 19, 2014, and December 17, 2017. Ethical approval was obtained by each site in accordance with their local regulations (https://www.center-tbi.eu/project/ethical-approval). Informed consent was obtained from all study participants or their legal representatives/relatives according to the local regulations of each center. Clinical data was accessed via the Neurobot platform (RRID/SCR_017004, core data, version 3.0; International Neuroinformatics Coordinating Facility; released November 24, 2020).

Patients were included in the analysis for this study if they were aged ≥8 years, had blood samples taken within 24 h of injury analyzed for lipidomics, and had an MRI scan performed within four weeks of injury. For patients who had multiple MRI scans, the earliest one was used. At each site which collected MRI data healthy controls (104 in total) were also scanned using identical sequences to enable harmomisation of DTI as well as comparison of quantitative metrics with patients. These healthy controls did not have blood sampling performed (Table 1). All severities of TBI were included and patients who had serious preexisting neurological disorders were excluded. The population of the CENTER-TBI study is predominantly Caucasian, and although this is reflective of the underlying European population served and similar to other large European based observational studies, this serves as an important limitation. Looking at the participants in this specific analysis all self-identified as Caucasian except for two who seld-identified as individuals of east Asian descent.

In total there were 103 patients that had both an MRI scan and blood samples available for lipidomic analysis, where gender information can also be found (Table 1). A flowchart of all the analyses and data acquisition can be seen in Figure 1.

### Method details

#### Analysis of lipids

Single blood samples of the patients were drawn within 24 h of injury using gel-separator tubes for serum and centrifuged within 60 min. Serum was processed, aliquoted (8 × 0·5 mL, one freeze-thaw cycle), and stored at −80°C locally and at the central CENTER-TBI biobank (Pécs, Hungary)^44^ until shipment on dry ice to Örebro University, Sweden for analysis.

The lipidomic platform for data analysis in this study has been described in detail elsewhere.^10^ The analysis was performed with an adjusted version of the Folch procedure.^45^ The internal standards used, the calibration curves, the instrument description, and the sample analysis using ultra-high-performance liquid chromatography quadrupole time-of-flight mass spectrometry are the same as described previously.^10^ Shortly, the plasma samples were randomized 10 μL of serum was mixed with 10 μL 0.9% NaCl and extracted with 120 μL of CHCl_3_: MeOH (2:1, v/v) solvent mixture containing internal standard mixture (c = 2.5 μg/mL; 1,2-diheptadecanoyl-*sn*-glycero-3-phosphoethanolamine (PE(17:0/17:0)), N-heptadecanoyl-D-*erythro*-sphingosylphosphorylcholine (SM(d18:1/17:0)), N-heptadecanoyl-D-*erythro*-sphingosine (Cer(d18:1/17:0)), 1,2-diheptadecanoyl-*sn*-glycero-3-phosphocholine (PC(17:0/17:0)), 1-heptadecanoyl-2-hydroxy-*sn*-glycero-3-phosphocholine (LPC(17:0)) and 1-palmitoyl-d31-2-oleoyl-*sn*-glycero-3-phosphocholine (PC(16:0/d31/18:1)) and, triheptadecanoylglycerol (TG(17:0/17:0/17:0)). The samples were vortexed and let stand on the ice for 30 min before centrifugation (9400 rcf, 3 min). 60 μL of the lower layer of was collected and diluted with 60 μL of CHCl_3_: MeOH. The samples were kept at −80°C until analysis.

The samples were analyzed using an ultra-high-performance liquid chromatography quadrupole time-of-flight mass spectrometry (UHPLC-QTOFMS from Agilent Technologies; Santa Clara, CA, USA). The analysis was carried out on an ACQUITY UPLC BEH C18 column (2.1 mm × 100 mm, particle size 1.7 μm) by Waters (Milford, USA). Quality control was performed throughout the dataset by including blanks, pure standard samples, extracted standard samples and control plasma samples. The eluent system consisted of (A) 10 mM NH_4_Ac in H_2_O and 0.1% formic acid and (B) 10 mM NH_4_Ac in ACN: IPA (1:1) and 0.1% formic acid. The gradient was as follows: 0–2 min, 35% solvent B; 2–7 min, 80% solvent B; 7–14 min 100% solvent B. The flow rate was 0.4 mL/min.

Data were processed using the open source software package MZmine 2.53.^46^ The following steps were applied in this processing: (i) Mass detection with a noise level of 1000, (ii) Chromatogram builder with a minimum time span of 0.08 min, minimum height of 1000 and an m/z tolerance of 0.006 m/z or 10.0 ppm, (iii) Chromatogram deconvolution using the local minimum search algorithm with a 70% chromatographic threshold, 0.05 min minimum RT range, 5% minimum relative height, 1200 minimum absolute height, a minimum ration of peak top/edge of 1.2 and a peak duration range of 0.08–5.0, (iv), Isotopic peak grouper with an m/z tolerance of 5.0 ppm, RT tolerance of 0.05 min, maximum charge of 2 and with the most intense isotope set as the representative isotope, (v) Join aligner with an m/z tolerance of 0.009 or 10.0 ppm and a weight for of 2, an RT tolerance of 0.15 min and a weight of 1 and with no requirement of charge state or ID and no comparison of isotope pattern, (vi) Peak list row filter with a minimum of 10% of the samples (vii) Gap filling using the same RT and m/z range gap filler algorithm with an m/z tolerance of 0.009 m/z or 11.0 ppm, (vii) Identification of lipids using a custom database search with an m/z tolerance of 0.008 m/z or 8.0 ppm and an RT tolerance of 0.25 min. Identification of lipids was based on in house laboratory based on LC-MS/MS data on retention time and mass spectra. The identification was done with a custom database, with identification levels 1 and 2, i.e., based on authentic standard compounds (level 1) and based on MS/MS identification (level 2) based on Metabolomics Standards Initiative. Quality control was performed by analysing pooled quality control samples (with an aliquot pooled from each individual samples) together with the samples. In addition, a reference standard (NIST 1950 reference plasma), extracted blank samples and standards were analyzed as part of the quality control procedure. List of lipid standards is provided in Table S7.

In total, 201 known lipids were quantified that belong to the major lipidomic functional groups including ceramides (Cer), lysophosphatidylcholines (LPC), phosphatidylcholines (PC), sphingomyelins (SM), and triacylglycerols (TG).

#### Image acquisition, processing, and harmonization

Patients underwent head CT scan on admission (within 24 h) and further CT scans were performed if necessary.

MRI scans were acquired on nine 3T MRI scanners across eight sites, using study specific protocols the details of which can be found at: https://www.center-tbi.eu/project/mri-study-protocols.^47^ Sequences included volumetric T1-weighted MPRAGE (voxel size 1 mm), volumetric fluid-attenuated inversion recovery, T2-weighted, susceptibility-weighted imaging and DTI. Base values of DTI were 2-mm isotropic voxels, 32 noncollinear directions, and a b value of 1000 s/mm^2^.

The initial CT and MRI images were reported centrally for the visible presence of lesions according to the Common Data Element (CDE) scheme for TBI (https://commondataelements.ninds.nih.gov/).^48^^,^^49^ Patients were classified as having a clinically abnormal MRI when at least one intracranial lesion secondary to TBI was detected.

All images were processed on a TBI-specific pipeline.^50^ Images underwent neck cropping and were corrected for bias field inhomogeneity. Diffusion images were corrected for noise, artifacts (Gibbs, head motion and eddy currents)^51^^,^^52^^,^^53^^,^^54^ and inhomogeneities in the magnetic field.^55^ Diffusion tensors were fitted via weighted least-squares to generate fractional anisotropy (FA) and mean diffusivity (MD) maps using fsl (https://fsl.fmrib.ox.ac.uk). These were non-linearly co-registered using ANTS (http://stnava.github.io/ANTs/) to the JHU-ICBM FA template^24^ to extract mean FA and MD measures for the regions of interest (Table S1).

Differences in sites and scanners were corrected for using ComBat harmonization,^56^^,^^57^ a statistical technique to minimize unwanted scanner effect while preserving the biological variability, for the FA and MD sets (based on the healthy controls). The values of the volumetric data interest were normalized per patient, dividing each patient’s ROI values by their respective total brain volume.

#### Image classification based on clinical definitions

Patients underwent head CT scan on admission and further CT scans were performed if necessary. Patients underwent MRI either during or immediately after hospitalisation. Only the first CT and MRI scan were considered for this study. Centralised review of CT and MRI scans was performed according to the Common Data Element (CDE) classification for TBI^49^ (https://commondataelements.ninds.nih.gov/).

Both CT and conventional MRI images were categorised as either negative or positive. For both modalities, negativity means that no traumatic intracranial changes were detected in the central reading.

### Quantification and statistical analysis

All statistical analyses for this work were performed in the R statistical program version 4.1.2.

#### Classification models

We investigated whether lipidomic changes could classify whether intracranial lesions were detected on the MRI (MRI positive) or not (MRI negative) in both the full set of patients (CT positive and negative, *n* = 102), and as a secondary analysis in patients with no abnormalities on CT (*n* = 45).

A filtering process was applied to find a subset of lipids that had the highest association with the MRI findings. Two separate analyses were run, the first using a Welch t-test to find the lipids with the highest abilities to separate MRI positive/negative based on the *p*-values and the second based on a random forest model with the same task. For each of the algorithms, the top 30 lipids associated with the MRI findings were selected, and the intersect of these two results provided the final subset of lipids for further analysis.

The subset of lipids was used as predictors in penalized logistic regression models to assess their discriminatory ability. Performance was assessed in a test set following the model fit to a training set (70%–30% split) where the subset of lipids was selected from the training set. Two penalized models were selected lasso and ridge regression, and each set of the process was repeated 100 times (data split, subset selection, model fit on training set, evaluation on the testing set), and the results of the performance for each penalized model were aggregated.

The penalized models were also run on the full set of lipids to compare the performance between them and the reduced set, and to validate the filtering process. Those analyses yielded eight under the curve values, four for the MRI positivity/negativity discrimination on the full set of patients (CT positive and negative) and four for the MRI positivity/negativity discrimination for the subset of patients with negative CT.

#### Frequency matrices

A correlation analysis of the frequencies of the relationships was performed for the quantitative diffusivity metrics (FA and MD) features to determine which ROIs were the most frequently correlated with the lipids; and to determine if there was a topological association between the brain and the lipid concentrations. For each lipid/ROI combination the Pearson correlation was calculated together with the corresponding *p*-values. Since the lipids were log transformed and standardized before analyzing it was deemed that Pearson correlation was an appropriate metric. The correlations that were not significant after Holm correction were filtered out, through the *rcorr.adjust* function in the *RcmdrMisc* package. The final table had the 201 lipids and ROIs that had significant correlations with each lipid. The frequency table summed the ROIs to ascertain which have the most frequent significant associations with the different lipids.

Furthermore, for each ROI, it was determined if it was overall positively or negatively associated with the lipid concentrations (based on the mean value of correlations). A frequency beanplot of the ROIs was created to show the level of the associations of the ROIs with the lipids and whether their values tend to increase or decrease in relation to the concentration values of the lipids.

#### Abnormalities at the individual patient level

A separate analysis was carried on the individual patients to investigate which ones had the highest level of abnormalities defined using FA and MD. For those patients with the highest levels of abnormalities (see below for definition) an analysis was performed to investigate whether patients who exhibited the most damage in the white matter tracts also exhibit differences in lipid concentrations on the functional group level.

To identify which patients had the highest irregularities a comparison analysis was performed with the images of the healthy control participants of the study. For each ROI, a linear regression model was first fit with ROI as dependent variable and sex and age (fitted as a second-degree polynomial) as predictors. The average values and a confidence internal (1 standard deviation from baseline) for each range was calculated for the healthy controls ROI. Then, controlled for age and sex, for a particular ROI, if both of the FA and MD values were outside the respective baseline range, the ROI value was designated as abnormal. The overall burden of injury was determined by calculating percentages of abnormal ROIs for each patient.

The percentage burden of injury of the patients was correlated to the 48 ROIs to see if any patterns are shown for different brain areas. For this analysis the raw concentration of the lipids within each group was added and used for a heatmap of concentration related to the burden of injury (Figure 3).

#### Abnormalities at the aggregate level

To determine if the ROIs that were prominent in the frequency of associations to the lipids were related to the the initial TBI location, that is whether the most frequent ROIs were the ones that were most affected in the injury, an abnormality frequency analysis of the ROIs was performed. In this analysis the ROIs of the patients with TBI were compared with the respective ROIs of the healthy controls that undertook the scans.

For this analysis, a two-sample Kolmogorov-Smirnov test was applied for each ROI separately for the FA and MD sets to test whether the different ROIs were different between the patients with TBI and healthy controls. The *p*-values were corrected for multiple testing with the false discovery rate method for each set separately. The average value of significant correlations was then extracted for each ROI and these were projected on a brain map visualization using the CARIMAS^58^ software (https://turkupetcentre.fi/carimas/). For comparison, the frequency of abnormalities of each ROI was also projected to visually evaluate if significant correlations of lipids to ROIs is related to burden of injury.

#### Network analysis

For the construction of the partial correlation networks, a similar process as the construction of the frequency matrices was applied. The tables constructed in this case were 48 × 20 for the FA and MD feature sets and 51 × 20 for the volumetric set. The significant correlations between the lipids and the ROIs of the three sets were filtered out and the frequency that each lipid appears in each table was summed up. The 40 lipids with the most correlations were then selected (20% of the total amount of lipids), when 25 are shown in the frequency matrices (Table 2). The aim of this analysis was to find the lipids to be included in the partial correlation network, while the analysis of the frequency matrices was intended to visualize the patterns of relation between the ROIs and the lipids (Figure 2). The frequency matrices can point to the ROIs that relate the most to the lipids (Tables S4–S6), while the partial correlation network can show how the lipids and the brain regions are related to each other (Figure 5). These two analyses complement each other and elucidate the different ways that the brain regions and the circulating lipidome connect following acute TBI.

For the partial correlation network, the library *qgraph* in R was used. The top 40 lipids for each feature set were used, together with the ROIs. For the FA and MD feature sets the brain was split in three parts, midline, left and right and the correlations were controlled with the inclusion of time elapsed between injury and blood sample draw, time between the injury and the MR scan, propofol administration and age. Only the significant partial correlations are shown on the network with an alpha level of 0.05 for all sets.

### Additional resources

URL: https://www.center-tbi.eu/.

Clinical trial number: registered at clinicaltrials.gov NCT02210221.
