# Supplementary material for: Serum lipidome associates with neuroimaging features in patients with traumatic brain injury
Source: iScience. 2024 Aug 3;27(9):110654. doi: 10.1016/j.isci.2024.110654 (PMC11381842; doi:10.1016/j.isci.2024.110654)

iScience, Volume *27*

Supplemental information

Serum lipidome associates with neuroimaging features in patients with traumatic brain injury

Ilias Thomas, Virginia F.J. Newcombe, Alex M. Dickens, Sophie Richter, Jussi P. Posti, Andrew I.R. Maas, Olli Tenovuo, Tuulia Hyötyläinen, András Büki, David K. Menon, Matej Orešič, and CENTER-TBI MR subgroup Participants and Investigators

Table S1. The white matter tract data as segmented into 48 regions ROIs. Related to **Figure 1**.

**ROI index**	**FA and MD ROIs**	FA TBI	FA controls	MD TBI	MD controls
1	Middle cerebellar peduncle	0.528888	0.536778	0.000738	0.000735
2	Pontine crossing tract (a part of MCP)	0.47378	0.490104	0.000685	0.000664
3	Genu of corpus callosum	0.5724	0.622675	0.000844	0.000789
4	Body of corpus callosum	0.599582	0.643787	0.00088	0.000816
5	Splenium of corpus callosum	0.657421	0.691585	0.000863	0.000806
6	Fornix (column and body of fornix)	0.407501	0.462412	0.00168	0.001518
7	Corticospinal tract R	0.482079	0.510867	0.000699	0.000663
8	Corticospinal tract L	0.492456	0.518737	0.000709	0.000695
9	Medial lemniscus R	0.500674	0.534889	0.000786	0.000743
10	Medial lemniscus L	0.509897	0.542327	0.000766	0.000731
11	Inferior cerebellar peduncle R	0.556272	0.567027	0.000723	0.000716
12	Inferior cerebellar peduncle L	0.550338	0.562073	0.000729	0.000716
13	Superior cerebellar peduncle R	0.556254	0.57301	0.001171	0.001135
14	Superior cerebellar peduncle L	0.56727	0.590985	0.00117	0.001132
15	Cerebral peduncle R	0.68498	0.719007	0.000719	0.00069
16	Cerebral peduncle L	0.650086	0.68211	0.000726	0.000702
17	Anterior limb of internal capsule R	0.512738	0.551426	0.000766	0.000706
18	Inferior cerebellar peduncle L	0.529302	0.564977	0.000726	0.000693
19	Posterior limb of internal capsule R	0.631891	0.651355	0.000701	0.00067
20	Posterior limb of internal capsule L	0.637122	0.651818	0.000694	0.000668
21	Retrolenticular part of internal capsule R	0.560078	0.580227	0.000793	0.000752
22	Retrolenticular part of internal capsule L	0.575139	0.598275	0.00079	0.000754
23	Anterior corona radiata R	0.394231	0.435473	0.000804	0.000745
24	Anterior corona radiata L	0.389957	0.424531	0.000793	0.000748
25	Superior corona radiata R	0.450936	0.470778	0.000733	0.000683
26	Superior corona radiata L	0.456739	0.474496	0.000735	0.00069
27	Posterior corona radiata R	0.444791	0.468217	0.000818	0.00076
28	Posterior corona radiata L	0.433549	0.452859	0.000804	0.000753
29	Posterior thalamic radiation (include optic radiation) R	0.529308	0.561984	0.000827	0.000786
30	Posterior thalamic radiation (include optic radiation) L	0.527377	0.554075	0.00085	0.000802
31	Sagittal stratum (include inferior	0.528658	0.554706	0.000849	0.000799

	longitidinal fasciculus and inferior fronto-occipital fasciculus) R				
32	Sagittal stratum (include inferior longitidinal fasciculus and inferior fronto-occipital fasciculus) L	0.523316	0.553662	0.000875	0.000817
33	External capsule R	0.420257	0.445138	0.000787	0.000746
34	External capsule L	0.420018	0.447592	0.000796	0.000754
35	Cingulum (cingulate gyrus) R	0.466003	0.4979	0.000763	0.000731
36	Cingulum (cingulate gyrus) L	0.488772	0.528886	0.000752	0.000729
37	Cingulum (hippocampus) R	0.440397	0.461297	0.00078	0.000751
38	Cingulum (hippocampus) L	0.439021	0.462332	0.00079	0.000763
39	Fornix (cres) / Stria terminalis (can not be resolved with current resolution) R	0.501186	0.54379	0.000979	0.000875
40	Fornix (cres) / Stria terminalis (can not be resolved with current resolution) L	0.517927	0.554867	0.000879	0.000814
41	Superior longitudinal fasciculus R	0.462544	0.486172	0.000744	0.000707
42	Superior longitudinal fasciculus L	0.460489	0.482067	0.000737	0.000697
43	Superior fronto-occipital fasciculus (could be a part of anterior internal capsule) R	0.406647	0.446624	0.000823	0.000714
44	Superior fronto-occipital fasciculus (could be a part of anterior internal capsule) L	0.420153	0.44579	0.00077	0.0007
45	Uncinate fasciculus R	0.452716	0.484732	0.000784	0.000763
46	Uncinate fasciculus L	0.44281	0.473387	0.000784	0.000747
47	Tapetum R	0.44119	0.486251	0.001269	0.001121
48	Tapetum L	0.445882	0.479221	0.001257	0.001151

Table S2. The volumetric data ROIs as segmented into 51 regions. Related to **Figure 1**.

ROI Index	Collapsed ROI name
1	BrainStem
2	CerebellarVermis
3	LeftAcumbens
4	LeftAmygdala
5	LeftBasalForebrain
6	LeftCaudate
7	LeftCerebellarWhite
8	LeftCerebellumGrey
9	LeftCerebralWhiteMatter
10	LeftDorsolateralFrontal
11	LeftFrontalPole
12	LeftFusiformGyrus
13	LeftHippocampalComplex
14	LeftInsula
15	LeftLateralParietalLobe
16	LeftMedialFrontal
17	LeftMedialParietalLobe
18	LeftOccipitalLobe
19	LeftOrbitofrontal
20	LeftPallidum
21	LeftPrecentral
22	LeftPutamen
23	LeftSubcallosal
24	LeftTemporalLobe
25	LeftTemporalPole
26	LeftThalamus
27	RightAcumbens
28	RightAmygdala
29	RightBasalForebrain
30	RightCaudate
31	RightCerebellarWhite
32	RightCerebellumGrey

33	RightCerebralWhiteMatter
34	RightDorsolateralFrontal
35	RightFrontalPole
36	RightFusiformGyrus
37	RightHippocampalComplex
38	RightInsula
39	RightLateralParietalLobe
40	RightMedialFrontal
41	RightMedialParietalLobe
42	RightOccipitalLobe
43	RightOrbitofrontal
44	RightPallidum
45	RightPrecentral
46	RightPutamen
47	RightSubcallosal
48	RightTemporalLobe
49	RightTemporalPole
50	RightThalamus
51	Ventricle

Table S3. Findings of the MRI classification performance of the lipids. The results of the ridge reduced model are shown. Related to **Table 2**.

	(1/0/NA)	AUC (CI)
MRI findings (n=102)	(62/40)	0.85 (0.71- 0.98)
Subset of patients with negative CT (n=45)	(11/33/1)	0.7 (0.37- 0.98)

Table S4. The FA ROIs that show the most correlations to the lipids together with the frequencies and the sign of the average correlations. Related to **Table 2**.

Frequency	Sign of correlation	Name
0.323	+ (positive)	Superior corona radiata L
0.209	+ (positive)	Superior cerebellar peduncle R
0.204	+ (positive)	Medial Lemniscus L
0.194	+ (positive)	Corticospinal L
0.184	+ (positive)	Superior longitudinal fasciculus L
0.159	+ (positive)	Posterior limb of internal capsule L
0.159	+ (positive)	Superior corona radiata R
0.154	+ (positive)	Retrolenticular part of internal capsule R
0.149	+ (positive)	Cerebral peduncle L
0.139	+ (positive)	Posterior limb of internal capsule R
0.124	+ (positive)	Inferior cerebellar peduncle L
0.124	+ (positive)	Posterior corona radiata L
0.114	+ (positive)	Anterior corona radiata L

Table S5. The MD ROIs that show the most correlations to the lipids together with the frequencies and the sign of the average correlations. Related to **Table 2**.

Frequency	Sign of correlation	Name
0.184	- (negative)	Corticospinal L
0.154	- (negative)	Fornix (cres) L
0.119	- (negative)	Superior longitudinal fasciculus L
0.1	- (negative)	Retrolenticular part of internal capsule R

Table S6. The volumetric ROIs that show the most correlations to the lipids together with the frequencies and the sign of the average correlations. Related to **Table 2**.

Frequency	Sign of correlation	Name
0.159	+ (positive)	RightAcumbens
0.129	- (negative)	LeftMedialParietalLobe
0.129	+ (positive)	RightSubcallosal
0.119	+ (positive)	LeftBasalForebrain
0.119	- (negative)	LeftFusiformGyrus
0.114	- (negative)	RightBasalForebrain
0.109	+ (positive)	LeftSubcallosal
0.104	+ (positive)	LeftFrontalPole

Table S7: Key resource table of the lipid standards. Related to **Table 2**.

REAGENT or RESOURCE	SOURCE	IDENTIFIER
Chemicals		
2-diheptadecanoyl-sn-glycero-3-phosphoethanolamine (PE(17:0/17:0))	Avanti Polar Lipids	Cat#830756
N-heptadecanoyl-D-erythro-sphingosylphosphorylcholine (SM(d18:1/17:0))	Avanti Polar Lipids	Cat#860585
1-stearoyl-2-hydroxy-sn-glycero-3-phosphocholine (LPC(18:0))	Avanti Polar Lipids	Cat#855775
2-diheptadecanoyl-sn-glycero-3-phosphocholine (PC(17:0/17:0))	Avanti Polar Lipids	Cat#850360
1-heptadecanoyl-2-hydroxy-sn-glycero-3-phosphocholine (LPC(17:0))	Avanti Polar Lipids	Cat#855676
2-Dioctadecanoyl- -sn-glycero-3-phosphocholine (PC(18:0/18:0))	Avanti Polar Lipids	Cat#850333
1-Hexadecanoyl-2-oleoyl-sn-glycero-3-phosphocholine (PC(16:0/18:1)	Avanti Polar Lipids	Cat#850457
1-(9Z-octadecenoyl)-sn-glycero-3-phosphoethanolamine (LPE(18:1))	Avanti Polar Lipids	Cat#850456
1-Palmitoyl-2-Hydroxy-sn-Glycero-3-Phosphatidylcholine (LPC(16:0))	Avanti Polar Lipids	Cat#846725
triheptadecanoylglycerol (TG(17:0/17:0/17:0))	Larodan	Cat#33-1700
trihexadecanoalglycerol (TG(16:0/16:0/16:0))	Larodan	Cat#33-1610
1-stearoyl-2-linoleoyl-sn-glycerol (DG(18:0/18:2))	Avanti Polar Lipids	Cat#855675
3-trioctadecanoylglycerol (TG(18:0/18:0/18:0))	Larodan	Cat#33-1810
3β-Hydroxy-5-cholestene-3-linoleate (ChoE(18:2))	Larodan	Cat#64-1802
1-hexadecyl-2-(9Z-octadecenoyl)-sn-glycero-3-phosphocholine (PC(16:0e/18:1(9Z)))	Avanti Polar Lipids	Cat#800817
1-(1Z-octadecanyl)-2-(9Z-octadecenoyl)- sn-glycero-3-phosphocholine (PC(18:0p/18:1(9Z)))	Avanti Polar Lipids	Cat#878112
1-oleoyl-2-hydroxy-sn-glycero-3-phosphocholine (LPC(18:1))	Larodan	Cat#38-1801
1-palmitoyl-2-oleoyl-sn-glycero-3-phosphoethanolamine (PE(16:0/18:1))	Avanti Polar Lipids	Cat#852467
3β-hydroxy-5-cholestene-3-stearate (ChoE(18:0))	Larodan	Cat#64-1800
1-palmitoyl-d31-2-oleoyl-sn-glycero-3-phosphocholine (PC(16:0/d31/18:1))	Avanti Polar Lipids	Cat#850757
2-diheptadecanoyl-sn-glycero-3-phosphoethanolamine (PE(17:0/17:0))	Avanti Polar Lipids	Cat#830756
N-heptadecanoyl-D-erythro-sphingosylphosphorylcholine (SM(d18:1/17:0))	Avanti Polar Lipids	Cat#860585

1-stearoyl-2-hydroxy-sn-glycero-3-phosphocholine (LPC(18:0))	Avanti Polar Lipids	Cat#855775

Figure S1. The regions of interest for the fractional anisotropy and mean diffusivity measures of the white matter tracts. Related to **Figures 2,3, and 4**.

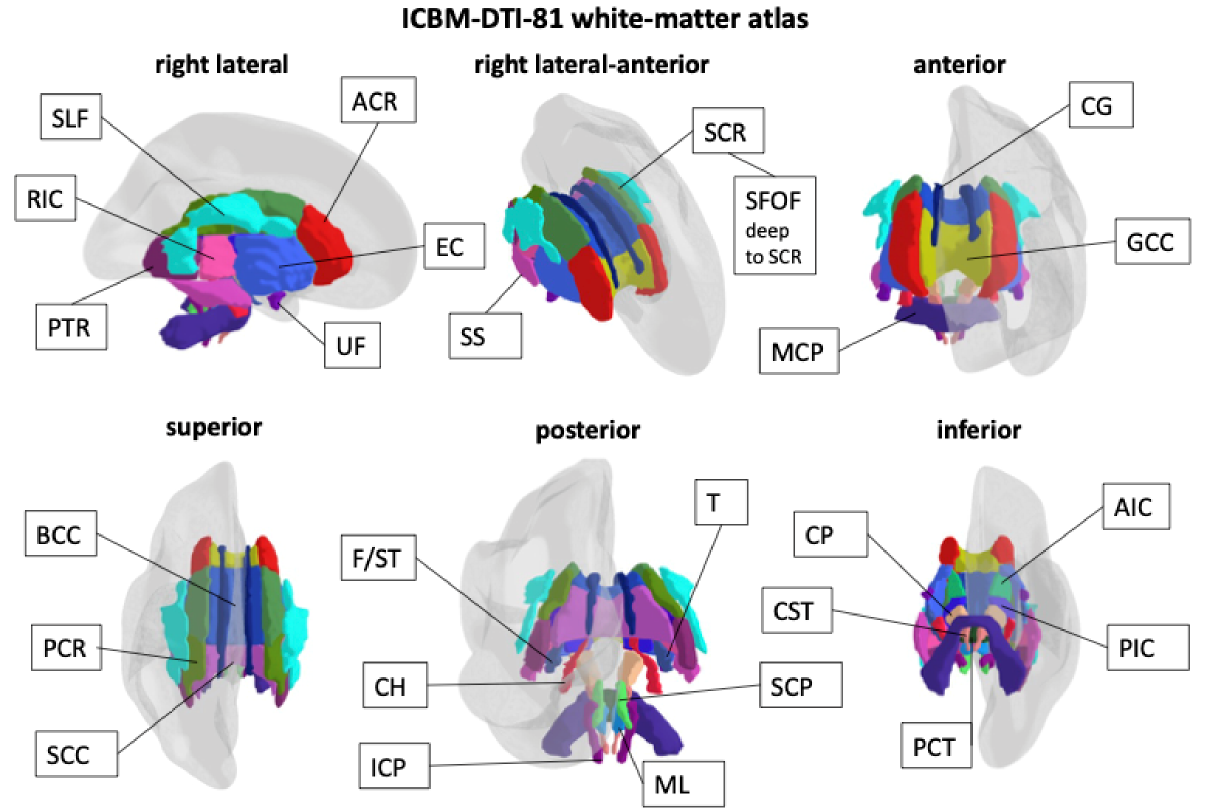

White matter labels of the ICBM-DTI-81 atlas in alphabetical order: ACR = Anterior corona radiata, AIC = Anterior limb of internal capsule, BCC = Corpus callosum (body), CP = Cerebral peduncle, CG = Cingulum (gyrus), CH = Cingulum (hippocampus), CST = Corticospinal tract, EC = External capsule, F/ST = Fornix (body and column) & Fornix (cres) / Stria terminalis, GCC = Corpus callosum (genu), ICP = Inferior cerebellar peduncle, ML = Medial lemniscus, MCP = Middle cerebellar peduncle, PCT = Pontine crossing tract, PCR = Posterior corona radiata, PIC = Posterior limb of internal capsule, PTR = Posterior thalamic radiation, RIC = Retrolenticular part of internal capsule, SS = Sagittal stratum, SCC = Corpus callosum (splenium), SCP = Superior cerebellar peduncle, SCR = Superior corona radiata, SFOF = Superior fronto-occipital fasciculus, SLF = Superior longitudinal fasciculus , T = Tapetum, UF = Uncinate fasciculus.

Figure S2. Beanpot of the correlation values of the volumetric ROIs to all the lipids. Predominantly negative correlations are seen which, on average, are at about -0.2. Related to **Figure 2**.

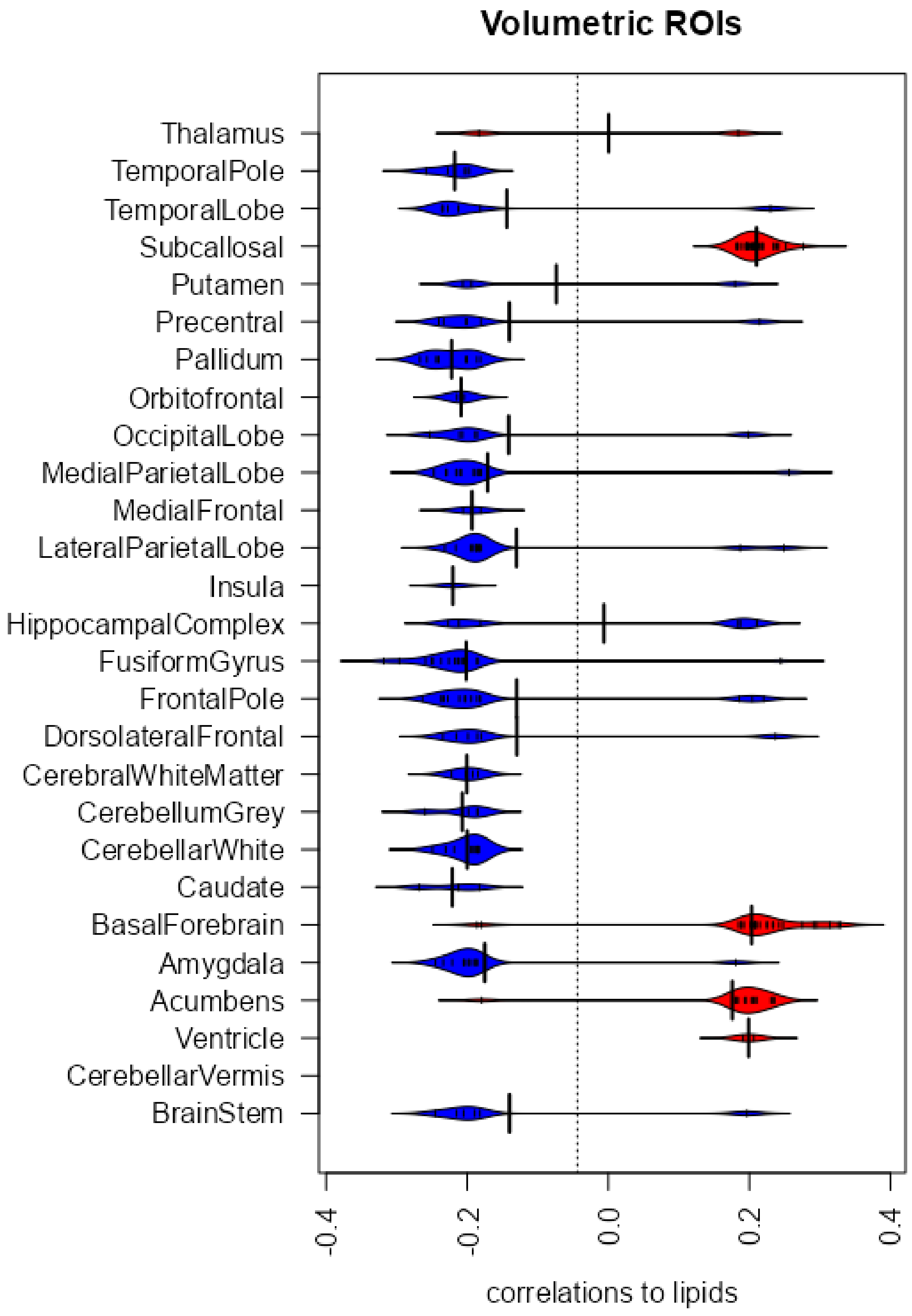

Supplement: Document S1. Figures S1, S2, and Tables S1–S7 [file mmc1.pdf]
